# Some Iterative Properties of $\left(F_{1},F_{2}\right)$-Chaos in Non-Autonomous Discrete Systems

**DOI:** 10.3390/e20030188

**Published:** 2018-03-12

**Authors:** Xiao Tang, Guanrong Chen, Tianxiu Lu

**Affiliations:** 1 School of Mathematical Sciences, Sichuan Normal University, Chengdu 610068, China; 2 Department of Electronic Engineering, City University of Hong Kong, Hong Kong SAR 999077, China; 3 School of Mathematics and Statistics, Sichuan University of Science and Engineering, Zigong 643000, China

**Keywords:** nonautonomous discrete system, Furstenberg family, scrambled sets, chaos, 37B55, 37D45, 54H20

## Abstract

This paper is concerned with invariance $\left(F_{1},F_{2}\right)$-scrambled sets under iterations. The main results are an extension of the compound invariance of Li–Yorke chaos and distributional chaos. New definitions of $\left(F_{1},F_{2}\right)$-scrambled sets in non-autonomous discrete systems are given. For a positive integer *k*, the properties P(k) and Q(k) of Furstenberg families are introduced. It is shown that, for any positive integer *k*, for any s∈[0,1], Furstenberg family $\bar{M}\left(s\right)$ has properties P(k) and Q(k), where $\bar{M}\left(s\right)$ denotes the family of all infinite subsets of $Z^{+}$ whose upper density is not less than *s*. Then, the following conclusion is obtained. *D* is an $\left(\bar{M}\left(s\right),\bar{M}\left(t\right)\right)$-scrambled set of $\left(X,f_{1,\infty }\right)$ if and only if *D* is an $\left(\bar{M}\left(s\right),\bar{M}\left(t\right)\right)$-scrambled set of $\left(X,f_{1,\infty }^{\left[m\right]}\right)$.

## 1. Introduction

Chaotic properties of a dynamical system have been extensively discussed since the introduction of the term chaos by Li and Yorke in 1975 [1] and Devaney in 1989 [2]. To describe some kind of unpredictability in the evolution of a dynamical system, other definitions of chaos have also been proposed, such as generic chaos [3], dense chaos [4], Li–Yorke sensitivity [5], and so on. An important generalization of Li–Yorke chaos is distributional chaos, which is given in 1994 by B. Schweizer and J. Smítal [6]. Then, theories related to scrambled sets are discussed extensively (see [7,8,9,10,11,12] and others). In 1997, the Furstenberg family was introduced by E. Akin [13]. J. Xiong, F. Tan described chaos with a couple of Furstenberg Families. $\left(F_{1},F_{2}\right)$-chaos has also been defined [14]. Moreover, F-sensitivity was given in [15] and shadowing properties were discussed in [16]. Most existing papers studied the chaoticity in autonomous discrete systems (X,f). However, if a sequence of perturbations to a system are described by different functions, then there are a sequence of maps to describe them, giving rise to non-autonomous systems. Non-autonomous discrete systems were precisely introduced in [17], in connection with non-autonomous difference equations (see [18,19] and some references therein).

Let (X,ρ) (briefly, *X*) be a compact metric space and consider a sequence of continuous maps $f_{n}:X\to X,n\in N$, denoted by $f_{1,\infty }=\left(f_{1},f_{2},\cdots \right)$. This sequence defines a non-autonomous discrete system $\left(X,f_{1,\infty }\right)$. The orbit of any point x∈X is given by the sequence $\left(f_{1}^{n}\left(x\right)\right)=Orb\left(x,f_{1,\infty }\right)$, where $f_{1}^{n}=f_{n}\circ \cdots \circ f_{1}$ for n≥1, and $f_{1}^{0}$ is the identity map.

For m∈N, define

$$g_{1}=f_{m}\circ \cdots \circ f_{1},\;g_{2}=f_{2m}\circ \cdots \circ f_{m+1},\;\cdots ,\;g_{p}=f_{pm}\circ \cdots \circ f_{(p-1)m+1},\;\cdots .$$

Call $\left(X,g_{1,\infty }\right)$ a compound system of $\left(X,f_{1,\infty }\right)$.

Also, denote $g_{1,\infty }$ by $f_{1,\infty }^{\left[m\right]}$ and denote $f_{n}^{k}=f_{n+k-1}\circ \cdots \circ f_{n}$ for n≥1. By [5], if ${\left(f_{n}\right)}_{n=1}^{\infty }$ converges uniformly to a map *f*. Then, for any m≥2(m∈N), the sequence ${\left(f_{n}^{n+m-1}\right)}_{n=1}^{\infty }$ converges uniformly to $f^{m}$.

In the present work, some notions relating to Furstenberg families and properties P(k), Q(k) are recalled in Section 2 and Section 3. Section 4 states some definitions about $\left(F_{1},F_{2}\right)$-chaos. In Section 5, it is proved that, under the conditions of property P(k) and positive shift-invariant, $f_{1,\infty }$ is $\left(F_{1},F_{2}\right)$-chaos (strong $\left(F_{1},F_{2}\right)$-chaos, strong F-chaos) implies $f_{1,\infty }^{\left[k\right]}$($k\in Z^{+}$) is $\left(F_{1},F_{2}\right)$-chaos (strong $\left(F_{1},F_{2}\right)$-chaos, strong F-chaos). If the conditions property Q(k) and negative shift-invariant both hold, the above conclusion can be inversed. As a conclusion, for arbitrary *s* and *t* in [0,1], for every $k\in Z^{+}$, $f_{1,\infty }$ and $f_{1,\infty }^{\left[k\right]}$ can share the same $\left(\bar{M}\left(s\right),\bar{M}\left(t\right)\right)$-scrambled set (Theorem 3).

In this paper, it is always assumed that all the maps $f_{n}$, n∈N, are surjective. It should be noted that this condition is needed by most papers dealing with this kind of system (for example, [20,21,22,23]). It is assumed that sequence ${\left(f_{n}\right)}_{n=1}^{\infty }$ converges uniformly. The aim of this paper is to investigate the $\left(F_{1},F_{2}\right)$-scrambled sets of $f_{1,\infty }$.

## 2. Furstenberg Families

Let P be the collection of all subsets of the positive integers set $Z^{+}=\left\{0,1,2,\ldots \right\}$. A collection F⊂P is called a Furstenberg family if it is hereditary upwards, i.e., $F_{1}\subset F_{2}$ and $F_{1}\in F$ imply $F_{2}\in F$. Obviously, the collection of all infinite subsets of $Z^{+}$ is a Furstenberg family, denoted by B.

Define the dual family kF of a Furstenberg family F by

$$kF=\left\{F\in P:Z^{+}-F\notin F\right\}=\{F\in P:F\cap F^{\prime }\neq \phi \;\mathrm{for}\;\mathrm{any}\;F^{\prime }\in F\}.$$

It is clear that kF is a Furstenberg family and k(kF)=F (see [13]).

For F∈P, $i\in Z^{+}$, let F−i={j−i≥0:j∈F} and F+i={j+i≥0:j∈F}. Furstenberg family F is positive shift-invariant if F+i∈F for every F∈F and any $i\in Z^{+}$. Furstenberg family F is negative shift-invariant if F−i∈F for every F∈F and any $i\in Z^{+}$. Furstenberg family F is shift-invariant if it is positive shift-invariant and negative shift-invariant.

The following shows a class of Furstenberg families which is related to upper density.

Let F⊂P. The upper density and the lower density of *F* are defined as follows:

$$\bar{\mu }\left(F\right)=\underset{n\to \infty }{lim sup}\frac{\#(F\cap \{0,1,\ldots ,\;n-1\})}{n},\;\;\underline{\mu }\left(F\right)=\underset{n\to \infty }{lim inf}\frac{\#(F\cap \{0,1,\ldots ,\;n-1\})}{n},$$

where #(A) denotes the cardinality of the set *A*.

For any *s* in [0,1], set $\bar{M}\left(s\right)=\left\{F\in B:\bar{\mu }\left(F\right)\geq s\right\}$.

**Proposition** **1.** For any s in [0,1], $\bar{M}\left(s\right)$ is shift-invariant Furstenberg family. And $\bar{M}\left(0\right)=B$.

**Proof.** 

(i) Let $F_{1},F_{2}\in \bar{M}\left(s\right),F_{1}\subset F_{2}$, then, ∀n∈N (where N={1,2,3,…}),
  $$\bar{\mu }\left(F_{1}\right)=\underset{n\to \infty }{lim sup}\frac{\#(F_{1}\cap \left\{0,1,\ldots ,\;n-1\right\})}{n}\leq \underset{n\to \infty }{lim sup}\frac{\#(F_{2}\cap \left\{0,1,\ldots ,\;n-1\right\})}{n}=\bar{\mu }\left(F_{2}\right)$$
  Thus, $F_{1}\in \bar{M}\left(s\right)$ (i.e., $\bar{\mu }\left(F_{1}\right)\geq s$) implies $F_{2}\in \bar{M}\left(s\right)$ (i.e., $\bar{\mu }\left(F_{1}\right)\geq s$). So, $\bar{M}\left(s\right)\left(\forall s\in \left[0,1\right]\right)$ are Furstenberg families.
(ii) Let $F\in \bar{M}\left(s\right)$, that is, $\bar{\mu }\left(F\right)=\underset{n\to \infty }{lim sup}\frac{\#(F\cap \{0,1,\ldots ,\;n-1\})}{n}\geq s$. Denote $F=\{t_{1},t_{2},\cdots \}$ (where $t_{k}\in Z^{+}$, $t_{k_{1}}<t_{k_{2}}\left(k_{1}<k_{2}\right)$), then $F+i=\{t_{1}+i,t_{2}+i,\cdots \}$ and $F-i=\left\{t_{k_{1}}-i,t_{k_{2}}-i,\cdots \right\}\left(t_{k_{j}}-i\geq 0\right)$ for any $i\in Z^{+}$.
  $$\underset{n\to \infty }{lim sup}\frac{\#((F+i)\cap \{0,1,\ldots ,\;n-1\})}{n}=\underset{n\to \infty }{lim sup}\frac{\#(\left\{t_{1}+i,t_{2}+i,\cdots \right\}\cap \left\{0,1,\ldots ,\;n-1\right\})}{n}$$
  $$=\underset{n\to \infty }{lim sup}\frac{\#(\left\{t_{1},t_{2},\cdots \right\}\cap \left\{0,1,\ldots ,\;n-1\right\})}{n}=\bar{\mu }\left(F\right)\geq s$$
  and
  $$\underset{n\to \infty }{lim sup}\frac{\#((F-i)\cap \{0,1,\ldots ,\;n-1\})}{n}\geq \underset{n\to \infty }{lim sup}\frac{\#(F\cap \{0,1,\ldots ,\;n-1\})-i}{n}=\bar{\mu }\left(F\right)\geq s$$
  So, $\bar{M}\left(s\right)$ is shift-invariant.
(iii) Obviously,
  $$\bar{M}\left(0\right)=\left\{F\in B:\bar{\mu }\left(F\right)\geq 0\right\}=\left\{F\in B:\underset{n\to \infty }{lim sup}\frac{\#(F\cap \{0,1,\ldots ,\;n-1\})}{n}\geq 0\right\}=B.$$
  This completes the proof.

☐

## 3. Properties P(k), Q(k) of Furstenberg Families

**Definition** **1.** *Let k be a positive integer and F be a Furstenberg family.*

*(1)*  For any F∈F, if there exists an integer j∈{0,1,⋯,k−1} such that $F_{k,j}=\left\{i\in Z^{+}:ki+j\in F\right\}\in F$, we say F have property P(k);
*(2)*  If $F_{k}=\left\{ki+j\in Z^{+}:j\in \left\{0,1,\cdots ,k-1\right\},i\in F\right\}\in F$, we say F have property Q(k).

The following proposition is given by [24]. For completeness, we give the proofs.

**Proposition** **2.** For any s∈[0,1] and any $k\in Z^{+}$, $\bar{M}\left(s\right)$ have properties P(k) and Q(k).

**Proof.** 

(1) If k=1, $\forall F\in \bar{M}\left(s\right)$, $F_{1,0}=\left\{i\in Z^{+}:i\in F\right\}=F$, i.e., there exists an integer j=0 such that $F_{k,j}\in \bar{M}\left(s\right)$. The following will discuss the case k>1.
  If s=0, $\bar{M}\left(0\right)=B$. ∀F∈B, $\forall k\in Z^{+}$, obviously, there exist j∈{0,1,…,k−1} such that $F_{k,j}\in B$.
  If 0<s≤1, suppose properties P(k) does not hold. Then there exists a $F\in \bar{M}\left(s\right)$ such that $\bar{\mu }\left(F_{k,j}\right)<s$ for every j∈{0,1,…,k−1}.
  For any j∈{0,1,…,k−1}, put $\varepsilon_{j}>0$ which satisfied $\bar{\mu }\left(F_{k,j}\right)<s-\varepsilon_{j}$. One can find a sufficiently large number *N* such that, n≥N, ${\#}_{n}\left(F_{k,j}\right)<n\left(s-\varepsilon_{j}\right)$ (where ${\#}_{n}\left(F_{k,j}\right)$ denotes the cardinality of the set $F_{k,j}\cap \left\{0,1,\ldots ,\;n-1\right\}$). Then ${\#}_{n}\left(F_{k,j}^{c}\right)>n-n\left(s-\varepsilon_{j}\right)$, where $F_{k,j}^{c}$ denotes the complementary set of $F_{k,j}$.
  Give an integer $m=kn+l_{m}>kN$, $l_{m}\in \left\{0,1,\ldots ,\;k-1\right\}$. By the definition of $F_{k,j}$, ki+j∉F if $i\notin F_{k,j}$. And $ki_{1}+j_{1}\neq ki_{2}+j_{2}$ if $i_{1},i_{2}\in \left\{0,1,\ldots ,\;n-1\right\}$, $j_{1},j_{2}\in \left\{0,1,\ldots ,\;k-1\right\}$ and $j_{1}\neq j_{2}$. Then
  $${\#}_{m}\left(F^{c}\right)\geq \sum_{j=0}^{k-1}{\#}_{n}\left(F_{k,j}^{c}\right)>\sum_{j=0}^{k-1}\left(n-n\left(s-\varepsilon_{j}\right)\right).$$
  So,
  $${\#}_{m}\left(F\right)<m-\sum_{j=0}^{k-1}\left(n-n\left(s-\varepsilon_{j}\right)\right).$$
  Put $\varepsilon =min\{\varepsilon_{j}:j=0,1,\ldots ,\;k-1\}$, then
  $$\bar{\mu }\left(F\right)=\underset{n\to \infty }{lim sup}\frac{{\#}_{m}\left(F\right)}{m}\leq \lim_{n\to \infty }\frac{m-\sum_{j=0}^{k-1}\left(n-n\left(s-\varepsilon_{j}\right)\right)}{m}\leq \lim_{n\to \infty }\frac{m-k(n-n(s-\varepsilon ))}{m}$$
  $$=\lim_{n\to \infty }\frac{kn+l_{m}-kn+kn\left(s-\varepsilon \right)}{kn+l_{m}}=s-\varepsilon <s$$
  This contradicts to $\bar{\mu }\left(F\right)\geq s$.
(2) Similarly, just consider the case k>1, 0<s≤1.
  Suppose properties Q(k) does not hold. Then there exists an integer $F\in \bar{M}\left(s\right)$ such that $\bar{\mu }\left(F_{k}\right)<s$. Put ε>0 which satisfied $\bar{\mu }\left(F_{k}\right)<s-\varepsilon$. One can find a sufficiently large number *N* such that, m≥N, ${\#}_{m}\left(F_{k}\right)<m\left(s-\varepsilon \right)$. Give a $m=kn+l_{m}>kN\left(m\geq N\right)$, $l_{m}\in \left\{0,1,\ldots ,\;k-1\right\}$. By the definition of $F_{k}$, $ki+j\in F_{k}\left(j\in \left\{0,1,\ldots ,\;k-1\right\}\right)$ if i∈F. And $ki_{1}+j_{1}\neq ki_{2}+j_{2}$ if $i_{1}\neq i_{2}$ and $j_{1},j_{2}\in \left\{0,1,\ldots ,\;k-1\right\}$. Then
  $$k\left({\#}_{n}\left(F\right)\right)\leq {\#}_{m}\left(F_{k}\right)<m\left(s-\varepsilon \right).$$
  So,
  $$\bar{\mu }\left(F\right)\leq \lim_{n\to \infty }\frac{m(s-\varepsilon )}{kn}=\lim_{n\to \infty }\frac{\left(kn+l_{m}\right)\left(s-\varepsilon \right)}{kn}=s-\varepsilon \leq s.$$
  This contradicts to $\bar{\mu }\left(F\right)\geq s$.
  This completes the proof.

☐

## 4. $\left(F_{1},F_{2}\right)$-Chaos in Non-Autonomous Systems

Now, we state the definition of $\left(F_{1},F_{2}\right)$-chaos in nonautonomous systems.

**Definition** **2.** *Let (X,ρ) be a compact metric space, $F_{1}$ and $F_{2}$ are two Furstenberg families. D⊂X is called a $\left(F_{1},F_{2}\right)$-scrambled set of $\left(X,f_{1,\infty }\right)$ (briefly, $f_{1,\infty }$), if ∀x≠y∈D, the following two conditions are satisfied:*

*(i)*  ∀t>0, $\left\{n\in N:\rho (f_{1}^{n}\left(x\right),f_{1}^{n}\left(y\right))<t\right\}\in F_{1}$;
*(ii)*  ∃δ>0, $\left\{n\in N:\rho (f_{1}^{n}\left(x\right),f_{1}^{n}\left(y\right))>\delta \right\}\in F_{2}$.

The pair (x,y) which satisfies the above two conditions is called an $\left(F_{1},F_{2}\right)$-scrambled pair of $f_{1,\infty }$.

*$f_{1,\infty }$ is said to be $\left(F_{1},F_{2}\right)$-chaotic if there exists an uncountable $\left(F_{1},F_{2}\right)$-scrambled set of $f_{1,\infty }$. If $F_{1}=F_{2}=F$, $f_{1,\infty }$ is said to be F-chaotic and (x,y) is an F-scrambled pair. $f_{1,\infty }$ is said to be strong $\left(F_{1},F_{2}\right)$-chaotic if there are some δ>0 and an uncountable subset D⊂X such that for any x,y∈D with x≠y, the following two conditions holds:*

*(i)*  $\left\{n\in N:\rho (f_{1}^{n}\left(x\right),f_{1}^{n}\left(y\right))<t\right\}\in F_{1}$ for all t>0;
*(ii)*  $\left\{n\in N:\rho (f_{1}^{n}\left(x\right),f_{1}^{n}\left(y\right))>\delta \right\}\in F_{2}$.

$f_{1,\infty }$ is said to be strong F-chaos if it is strong $\left(F_{1},F_{2}\right)$-chaotic and $F_{1}=F_{2}=F$.

Let us recall the definitions of Li-Yorke chaos and distributional chaos in non-autonomous systems (see [25,26]).

**Definition** **3.** *Assume that $\left(X,f_{1,\infty }\right)$ is a non-autonomous discrete system. If x,y∈X with x≠y, (x,y) is called a Li–Yorke pair if*

$$\underset{n\to \infty }{lim sup}\rho \left(f_{1}^{n}\left(x\right),f_{1}^{n}\left(y\right)\right)>0\;\;\;and\;\;\;\underset{n\to \infty }{lim inf}\rho \left(f_{1}^{n}\left(x\right),f_{1}^{n}\left(y\right)\right)=0.$$

The set D⊂X is called a Li–Yorke scrambled set if all points x,y∈D with x≠y, (x,y) is a Li–Yorke pair. $f_{1,\infty }$ is Li–Yorke chaotic if X contains an uncountable Li–Yorke scrambled set.

Assume that $\left(X,f_{1,\infty }\right)$ is a non-autonomous discrete system. For any pair of points x,y∈X, define the upper and lower (distance) distributional functions generated by $f_{1,\infty }$ as

$$F_{xy}^{*}\left(t,f_{1,\infty }\right)=\underset{n\to \infty }{lim sup}\frac{1}{n}\sum_{i=1}^{n}\chi_{\left[0,t\right)}\left(\rho \left(f_{1}^{i}\left(x\right),f_{1}^{i}\left(y\right)\right)\right)$$

and

$$F_{xy}\left(t,f_{1,\infty }\right)=\underset{n\to \infty }{lim inf}\frac{1}{n}\sum_{i=1}^{n}\chi_{\left[0,\delta \right)}\left(\rho \left(f_{1}^{i}\left(x\right),f_{1}^{i}\left(y\right)\right)\right)$$

respectively. Where $\chi_{\left[0,t\right)}$ is the characteristic function of the set [0,t), i.e., $\chi_{\left[0,t\right)}\left(a\right)=1$ when a∈[0,t) or $\chi_{\left[0,t\right)}\left(a\right)=0$ when a∉[0,t).

**Definition** **4.** $f_{1,\infty }$ is distributionally chaotic if exists an uncountable subset D⊂X such that for any pair of distinct points x,y∈D, we have that $F_{xy}^{*}\left(t,f_{1,\infty }\right)=1$ for all t>0 and $F_{xy}\left(t,f_{1,\infty }\right)=0$ for some δ>0.

The set D is a distributionally scrambled set and the pair (x,y) a distributionally chaotic pair.

It is not difficult to obtain that the pair (x,y) is a $\left(\bar{M}\left(0\right),\bar{M}\left(0\right)\right)$-scrambled pair if and only if (x,y) is a Li–Yorke scrambled pair, and the pair (x,y) is a $\left(\bar{M}\left(1\right),\bar{M}\left(1\right)\right)$-scrambled pair if and only if (x,y) is a distributionally scrambled pair. In fact,

$$\bar{M}\left(0\right)=B,\bar{M}\left(1\right)=\left\{F\in B:\underset{n\to \infty }{lim sup}\frac{\#(F\cap \{1,2,\ldots ,\;n\})}{n}=1\right\}.$$

Then, $\left\{n\in N:\rho \left(f_{1}^{n}\left(x\right),f_{1}^{n}\left(y\right)\right)<t\right\}\in \bar{M}\left(0\right)$ for any t>0 and $\left\{n\in N:\rho \left(f_{1}^{n}\left(x\right),f_{1}^{n}\left(y\right)\right)>\delta \right\}\in \bar{M}\left(0\right)$ for some δ>0 is equivalent to that $\underset{n\to \infty }{lim sup}\rho \left(f_{1}^{n}\left(x\right),f_{1}^{n}\left(y\right)\right)>0$ and $\underset{n\to \infty }{lim inf}\rho \left(f_{1}^{n}\left(x\right),f_{1}^{n}\left(y\right)\right)=0$. $\left\{n\in N:\rho \left(f_{1}^{n}\left(x\right),f_{1}^{n}\left(y\right)\right)<t\right\}\in \bar{M}\left(1\right)$ for any t>0 and $\left\{n\in N:\rho \left(f_{1}^{n}\left(x\right),f_{1}^{n}\left(y\right)\right)>\delta \right\}\in \bar{M}\left(1\right)$ for some δ>0 is equivalent to that $F_{xy}^{*}\left(t,f_{1,\infty }\right)=1$ and $F_{xy}\left(\delta ,f_{1,\infty }\right)=0$.

Hence, $\left(\bar{M}\left(0\right),\bar{M}\left(0\right)\right)$-chaos is Li–Yorke chaos and $\left(\bar{M}\left(1\right),\bar{M}\left(1\right)\right)$-chaos is distributional Chaos.

## 5. Main Results

**Theorem** **1.** Let $F_{1}$ and $F_{2}$ are two Furstenberg families with property P(k), where k is a positive integer. $F_{1}$ is positive shift-invariant. If the system $\left(X,f_{1,\infty }\right)$ is $\left(F_{1},F_{2}\right)$-chaos, then the system $\left(X,f_{1,\infty }^{\left[k\right]}\right)$ is $\left(F_{1},F_{2}\right)$-chaos too.

**Proof.**  If *D* is an $\left(F_{1},F_{2}\right)$-scrambled set of $f_{1,\infty }$, the following proves that *D* is an $\left(F_{1},F_{2}\right)$-scrambled set of $f_{1,\infty }^{\left[k\right]}$.

(i) Since *X* is compact and $f_{i}\left(i\in N\right)$ are continuous, then, for any j∈{1,2,…,k−1}, $f_{s_{1}},\ldots ,\;f_{s_{k-j}}$ are uniformly continuous (where $f_{s_{1}},\ldots ,\;f_{s_{k-j}}$ are freely chosen from the sequence $f_{i}\left(i\in N\right)$). That is, for any δ>0, there exists a $\delta^{*}>0$, ∀a,b∈X, $\rho \left(a,b\right)<\delta^{*}$ implies $\rho (f_{s_{k-j}}\circ \cdots \circ f_{s_{1}}\left(a\right),f_{s_{k-j}}\circ \cdots \circ f_{s_{1}}\left(b\right))<\delta$ (j=1,2,…,k−1).
  Since *D* is an $\left(F_{1},F_{2}\right)$-scrambled set of $f_{1,\infty }$, then, ∀x≠y∈D, for the above $\delta^{*}$, we have
  $$F=\left\{n\in N:\rho \left(f_{1}^{n}\left(x\right),f_{1}^{n}\left(y\right)\right)<\delta^{*}\right\}\in F_{1}.$$
  And because $F_{1}$ have property P(k), there exists some j∈{1,2,…,k−1} such that
  $$F_{k,j}=\left\{i\in Z^{+}:ki+j\in F\right\}=\left\{i\in Z^{+}:\rho \left(f_{1}^{ki+j}\left(x\right),f_{1}^{ki+j}\left(y\right)\right)<\delta^{*}\right\}\in F_{1}.$$
  By the selection of $\delta^{*}$, we put $s_{r}=ki+j+r\left(r=1,2,\ldots ,\;k-j\right)$, then
  $$F_{k,j}\subset \left\{i\in Z^{+}:\rho \left(f_{1}^{ki+j+k-j}\left(x\right),f_{1}^{ki+j+k-j}\left(y\right)\right)<\delta \right\}=\left\{i\in Z^{+}:\rho \left(f_{1}^{k(i+1)}\left(x\right),f_{1}^{k(i+1)}\left(y\right)\right)<\delta \right\}.$$
  Write $F_{k,j}+1=\left\{i+1:i\in Z^{+},ki+j\in F_{1}\right\}\left(\forall j=1,2,\ldots ,\;k-1\right)$, then $F_{k,j}+1\subset \left\{i\in Z^{+}:\rho \left(f_{1}^{ki}\left(x\right),f_{1}^{ki}\left(y\right)\right)<\delta \right\}$.
  By the positive shift-invariant of $F_{1}$ and $F_{k,j}\in F_{1}$, we have $F_{k,j}+1\in F_{1}$. And with the hereditary upwards of $F_{1}$, for any x,y∈D:x≠y, ∀δ>0, $\left\{i\in Z^{+}:\rho \left(f_{1}^{ki}\left(x\right),f_{1}^{ki}\left(y\right)\right)<\delta \right\}\in F_{1}$.
(ii) Since *D* is a $\left(F_{1},F_{2}\right)$-scrambled set of $f_{1,\infty }$, then, for the above x,y∈D(x≠y), $\exists \varepsilon^{*}>0$, such that $E=\left\{n\in Z^{+}:\rho \left(f_{1}^{n}\left(x\right),f_{1}^{n}\left(y\right)\right)>\varepsilon^{*}\right\}\in F_{2}$. And because $F_{2}$ have property P(k), then, there exists some j∈{1,2,…,k−1} such that
  $$E_{k,j}=\left\{i\in Z^{+}:ki+j\in E\right\}=\left\{i\in Z^{+}:\rho \left(f_{1}^{ki+j}\left(x\right),f_{1}^{ki+j}\left(y\right)\right)>\varepsilon^{*}\right\}\in F_{2}.$$
  *X* is compact and $f_{i}\left(i\in N\right)$ are continuous, then, for any j∈{1,2,…,k−1}, $f_{s_{1}},\ldots ,\;f_{s_{j}}$ are uniformly continuous (where $f_{s_{1}},\ldots ,\;f_{s_{j}}$ are freely chosen from the sequence $f_{i}\left(i\in N\right)$). For the above $\varepsilon^{*}>0$, ∃ε>0, ∀p,q∈X satisfied ρ(p,q)≤ε, inequality $\rho \left(f_{s_{j}}\circ \cdots \circ f_{s_{1}}\left(p\right),f_{s_{j}}\circ \cdots \circ f_{s_{1}}\left(q\right)\right)\leq \varepsilon^{*}$ holds.
  The following will prove that $\left\{i\in Z^{+}:\rho \left(f_{1}^{ki}\left(x\right),f_{1}^{ki}\left(y\right)\right)>\varepsilon \right\}\in F_{2}$.
  Suppose $\left\{i\in Z^{+}:\rho \left(f_{1}^{ki}\left(x\right),f_{1}^{ki}\left(y\right)\right)>\varepsilon \right\}\notin F_{2}$, then
  $$Z^{+}-\left\{i\in Z^{+}:\rho \left(f_{1}^{ki}\left(x\right),f_{1}^{ki}\left(y\right)\right)>\varepsilon \right\}=\left\{i\in Z^{+}:\rho \left(f_{1}^{ki}\left(x\right),f_{1}^{ki}\left(y\right)\right)\leq \varepsilon \right\}\in kF_{2}.$$
  By the selection of $\varepsilon^{*}$, we put $s_{r}=ki+r\left(r=1,2,\ldots ,\;j\right)$, then
  $$\left\{i\in Z^{+}:\rho \left(f_{1}^{ki+j}\left(x\right),f_{1}^{ki+j}\left(y\right)\right)\leq \varepsilon^{*}\right\}\in kF_{2}.$$
  So,
  $$\left\{i\in Z^{+}:\rho \left(f_{1}^{ki+j}\left(x\right),f_{1}^{ki+j}\left(y\right)\right)>\varepsilon^{*}\right\}\notin kF_{2},$$
  This contradicts $E_{k,j}\in F_{2}$.
  Hence, for x≠y∈D in (i), there exists a ε>0 such that $\left\{i\in Z^{+}:\rho \left(f_{1}^{ki}\left(x\right),f_{1}^{ki}\left(y\right)\right)>\varepsilon \right\}\in F_{2}$.
  Combining with (i) and (ii), $f_{1,\infty }^{\left[k\right]}$ is $\left(F_{1},F_{2}\right)$-chaos.
  This completes the proof.

☐

**Theorem** **2.** Let $F_{1}$ and $F_{2}$ are two Furstenberg families with property Q(k), where k is a positive integer. $F_{2}$ is negative shift-invariant. If the system $\left(X,f_{1,\infty }^{\left[k\right]}\right)$ is $\left(F_{1},F_{2}\right)$-chaos, then the system $\left(X,f_{1,\infty }\right)$ is $\left(F_{1},F_{2}\right)$-chaos too.

**Proof.**  If *D* is a $\left(F_{1},F_{2}\right)$-scrambled set of $f_{1,\infty }^{\left[k\right]}$, the following prove that *D* is a $\left(F_{1},F_{2}\right)$-scrambled set of $f_{1,\infty }$.

(i) Similar to Theorem 1, for any j∈{1,2,…,k−1}, $f_{s_{1}},\ldots ,\;f_{s_{j}}$ are uniformly continuous (where $f_{s_{1}},\ldots ,\;f_{s_{j}}$ are freely chosen from the sequence $f_{i}\left(i\in N\right)$). That is, for any δ>0, there exists a $\delta^{*}>0$, ∀a,b∈X, $\rho \left(a,b\right)<\delta^{*}$ implies $\rho (f_{s_{j}}\circ \cdots \circ f_{s_{1}}\left(a\right),f_{s_{j}}\circ \cdots \circ f_{s_{1}}\left(b\right))<\delta$ (j=1,2,…,k−1).
  For any pair of distinct points x,y∈D, for the above $\delta^{*}$, one has
  $$F=\left\{n\in Z^{+}:\rho \left(f_{1}^{kn}\left(x\right),f_{1}^{kn}\left(y\right)\right)<\delta^{*}\right\}\in F_{1}.$$
  By the selection of $\delta^{*}$, for ∀n∈F, ∀j∈{1,2,⋯,k−1}, put $s_{r}=ki+j+r\left(r=1,2,\ldots ,\;j\right)$, then $\rho (f_{1}^{kn+j}\left(x\right),f_{1}^{kn+j}\left(y\right))<\delta$. And because $F_{1}$ have property Q(k), then
  $$F_{k}=\left\{kn+j\in Z^{+}:j=1,2,\ldots ,\;k-1,n\in F\right\}\in F_{1}.$$
  Notice that $F_{k}\subset \left\{m\in Z^{+}:\rho \left(f_{1}^{m}\left(x\right),f_{1}^{m}\left(y\right)\right)<\delta \right\}$, then $\left\{m\in Z^{+}:\rho \left(f_{1}^{m}\left(x\right),f_{1}^{m}\left(y\right)\right)<\delta \right\}\in F_{1}$.
(ii) Since *D* is an $\left(F_{1},F_{2}\right)$-scrambled set of $f_{1,\infty }^{\left[k\right]}$, then, for the above x,y∈D(x≠y), there exist $\varepsilon^{*}>0$, such that $E=\left\{n\in Z^{+}:\rho \left(f_{1}^{kn}\left(x\right),f_{1}^{kn}\left(y\right)\right)>\varepsilon^{*}\right\}\in F_{2}$.
  For any j∈{1,2,…,k−1}, $f_{s_{1}},\ldots ,\;f_{s_{j}}$ are uniformly continuous (where $f_{s_{1}},\ldots ,\;f_{s_{j}}$ are freely chosen from the sequence $f_{i}\left(i\in N\right)$), then, for the above $\varepsilon^{*}>0$, there exist ε>0 such that ρ(p,q)<ε(p,q∈X) implies $\rho \left(f_{s_{j}}\circ \cdots \circ f_{s_{1}}\left(p\right),f_{s_{j}}\circ \cdots \circ f_{s_{1}}\left(q\right)\right)\leq \varepsilon^{*}\left(j=1,2,\ldots ,\;k-1\right)$. That is, $\rho \left(f_{1}^{k}\left(p\right),f_{1}^{k}\left(q\right)\right)>\varepsilon^{*}\left(p,q\in X\right)$ implies $\rho (f_{1}^{j}\left(p\right),f_{1}^{j}\left(q\right))>\varepsilon \left(j=1,2,\ldots ,\;k-1\right)$.
  ∀n∈E, ∀j=1,2,…,k−1, put $s_{r}=k\left(n-1\right)+r\left(r=1,2,\ldots ,\;j\right)$, then
  $$\rho (f_{1}^{k(n-1)+j}\left(x\right),f_{1}^{k(n-1)+j}\left(y\right))>\varepsilon .$$
  Since $F_{2}$ is negative shift-invariant, then $E-1\in F_{2}$. And because $F_{2}$ have property Q(k), then ${\left(E-1\right)}_{k}\in F_{2}$, i.e., $\left\{k\left(n-1\right)+j\in Z^{+}:n-1\in E-1,j=1,2,\ldots ,\;k-1\right\}\in F_{2}$. Combining ${\left(E-1\right)}_{k}\subset \left\{m\in Z^{+}:\rho \left(f_{1}^{m}\left(x\right),f_{1}^{m}\left(y\right)\right)>\varepsilon \right\}$ with the hereditary upwards of $F_{2}$, we have $\left\{m\in Z^{+}:\rho \left(f_{1}^{m}\left(x\right),f_{1}^{m}\left(y\right)\right)>\varepsilon \right\}\in F_{2}$.
  By (i) and (ii), *D* is an $\left(F_{1},F_{2}\right)$-scrambled set of $f_{1,\infty }$.
  This completes the proof.

☐

Similarly, the following corollaries hold.

**Corollary** **1.** Let $F_{1}$ and $F_{2}$ are two Furstenberg families with property P(k), where k is a positive integer. $F_{1}$ is positive shift-invariant. If the system $\left(X,f_{1,\infty }\right)$ is F-chaos (strong $\left(F_{1},F_{2}\right)$-chaos, or strong F-chaos), then the system $\left(X,f_{1,\infty }^{\left[k\right]}\right)$ is F-chaos (strong $\left(F_{1},F_{2}\right)$-chaos, or strong F-chaos).

**Corollary** **2.** Let $F_{1}$ and $F_{2}$ are two Furstenberg families with property Q(k), where k is a positive integer. $F_{2}$ is negative shift-invariant. If the system $\left(X,f_{1,\infty }^{\left[k\right]}\right)$ is F-chaos (strong $\left(F_{1},F_{2}\right)$-chaos, or strong F-chaos), then the system $\left(X,f_{1,\infty }\right)$ is F-chaos (strong $\left(F_{1},F_{2}\right)$-chaos, or strong F-chaos).

Combining with Propositions 1 and 2, Theorems 1 and 2, and Corollarys 1 and 2, the following conclusions are obtained.

**Theorem** **3.** *Let s and t are arbitrary two numbers in [0,1], then*

*(1)*  If D is an $\left(\bar{M}\left(s\right),\bar{M}\left(t\right)\right)$-scrambled set (or strong $\left(\bar{M}\left(s\right),\bar{M}\left(t\right)\right)$-scrambled set) of $f_{1,\infty }$, then, for every $k\in Z^{+}$, D is an $\left(\bar{M}\left(s\right),\bar{M}\left(t\right)\right)$-scrambled set(or strong $\left(\bar{M}\left(s\right),\bar{M}\left(t\right)\right)$-scrambled set) of $f_{1,\infty }^{\left[k\right]}$.
*(2)*  For some positive integer k, if D is an $\left(\bar{M}\left(s\right),\bar{M}\left(t\right)\right)$-scrambled set (or strong $\left(\bar{M}\left(s\right),\bar{M}\left(t\right)\right)$-scrambled set) of $f_{1,\infty }^{\left[k\right]}$, then D is an $\left(\bar{M}\left(s\right),\bar{M}\left(t\right)\right)$-scrambled set (or strong $\left(\bar{M}\left(s\right),\bar{M}\left(t\right)\right)$-scrambled set) of $f_{1,\infty }$.

**Proof.** 

(1) By Proposition 1, $\bar{M}\left(s\right)$ is shift-invariant (obviously positive shift-invariant). And because $\bar{M}\left(s\right),\bar{M}\left(t\right)$ are two Furstenberg families with property P(k) (Proposition 2). Then, according to the proof of Theorem 1, if *D* is an $\left(\bar{M}\left(s\right),\bar{M}\left(t\right)\right)$-scrambled set of $f_{1,\infty }$, then, for every $k\in Z^{+}$, *D* is an $\left(\bar{M}\left(s\right),\bar{M}\left(t\right)\right)$-scrambled set of $f_{1,\infty }^{\left[k\right]}$.
(2) In the same way, (2) holds.
  This completes the proof.

☐

With the preparations in Section 4, we have

**Corollary** **3.**

*(1)*  If D is a Li–Yorke scrambled set (or distributionally scrambled set) of $f_{1,\infty }$, then, for every $k\in Z^{+}$, D is a Li–Yorke scrambled set (or distributionally scrambled set) of $f_{1,\infty }^{\left[k\right]}$.
*(2)*  For some positive integer k, if D is a Li–Yorke scrambled set (or distributionally scrambled set) of $f_{1,\infty }^{\left[k\right]}$, then, D is a Li–Yorke scrambled set (or distributionally scrambled set) of $f_{1,\infty }$.

**Remark** **1.** In the non-autonomous systems, the iterative properties of Li–Yorke chaos and distributional chaos are discussed in [25,26] before. The conclusions in Corollary 3 remains consistent with them.

This paper has presented several properties of $\left(F_{1},F_{2}\right)$-chaos, strong $\left(F_{1},F_{2}\right)$-chaos, and strong F-chaos. There are some other problems, such as generically F-chaos and F-sensitivity, to discuss. Moreover, property P(k) is closely related to congruence theory. Follow this line, one can consider other Furstenberg families which consist of number sets with some special characteristics.
